# Supplementary material for: Collaborative agglomeration level and spatial correlation of intercity manufacturing industry: An empirical study based on the cities of the Yangtze River Delta
Source: PLoS One. 2023 Nov 10;18(11):e0293645. doi: 10.1371/journal.pone.0293645 (PMC10637707; doi:10.1371/journal.pone.0293645)
Supplement: Attachment — Submitted filename: PONE-D-23-25180_reviewer.pdf [file pone.0293645.s002.pdf]

PLOS ONE

~~Research on the~~ Collaborative Agglomeration Level and Spatial Correlation of Intercity Manufacturing Industry: An Empirical Study Based on the Central Cities of the Yangtze River Delta

--Manuscript Draft--

**Manuscript Number:**	PONE-D-23-25180
**Article Type:**	Research Article
**Full Title:**	~~Research on the~~ Collaborative Agglomeration Level and Spatial Correlation of Intercity Manufacturing Industry: An Empirical Study Based on the ~~Central~~ Cities of the Yangtze River Delta
**Short Title:**	Research on the Collaborative Agglomeration Level and Spatial Correlation of Intercity Manufacturing Industry
**Corresponding Author:**	Zhu kai<br>Zhejiang University of Technology<br>CHINA
**Keywords:**	intercity; manufacturing; collaborative agglomeration; spatial correlation; Yangtze River Delta
**Abstract:**	Taking 27 cities in the ~~central area of the~~ Yangtze River Delta as an example, the time section data from 2009 to 2019 are selected, and the location entropy index and the modified E-G index are introduced to measure the collaborative agglomeration level of intercity manufacturing industry in the Yangtze River Delta. The spatial weight matrix is constructed based on the highway mileage between cities. Using Moran's index and Local Moran's index, this article analyzes the spatial correlation of the collaborative agglomeration level of intercity manufacturing industry in the Yangtze River Delta. The results show that: (1) The overall manufacturing industry agglomeration degree of cities in the central area of the Yangtze River Delta shows a fluctuating downward trend. The manufacturing industry agglomeration degree of Jiangsu and Zhejiang Province has decreased, that of most cities in Anhui Province has increased steadily. (2) The level of manufacturing industry collaborative agglomeration between Shanghai, Nanjing, Hangzhou and Jiangsu and Zhejiang cities showed a downward trend, while the level of collaborative agglomeration with Anhui cities increased steadily. The level of manufacturing industry collaborative agglomeration between Wuxi, Suzhou, Nanjing and cities with obvious advantages in manufacturing industry agglomeration degree shows an upward trend, while the level of collaborative agglomeration with other cities has declined. The collaborative agglomeration level of intercity manufacturing industry in Hefei has generally improved, especially the quality and depth of manufacturing industry collaborative agglomeration between Hefei and Anhui cities have significantly improved. (3) The regions with high level of intercity manufacturing industry collaborative agglomeration in the Yangtze River Delta are still mainly concentrated in Jiangsu and Zhejiang provinces. Although there has been a trend of westward transfer in the past decade, the overall change range is small, and the spatial correlation of the level of intercity manufacturing industry collaborative agglomeration shows a weakening trend as a whole, and the spatial differences continue to narrow.
**Order of Authors:**	Zhu kai
	Sun wan xiang
**Additional Information:**	
**Question**	**Response**
**Financial Disclosure**<br><br>Enter a financial disclosure statement that describes the sources of funding for the work included in this submission. Review the submission guidelines for detailed	The author(s) received no specific funding for this work.

Powered by Editorial Manager® and ProduXion Manager® from Aries Systems Corporation

requirements. View published research articles from *PLOS ONE* for specific examples.<br><br>This statement is required for submission and **will appear in the published article** if the submission is accepted. Please make sure it is accurate.<br><br>**Unfunded studies**<br>Enter: *The author(s) received no specific funding for this work.*<br><br>**Funded studies**<br>Enter a statement with the following details:<br>• Initials of the authors who received each award<br>• Grant numbers awarded to each author<br>• The full name of each funder<br>• URL of each funder website<br>• Did the sponsors or funders play any role in the study design, data collection and analysis, decision to publish, or preparation of the manuscript?<br>• **NO** - Include this sentence at the end of your statement: *The funders had no role in study design, data collection and analysis, decision to publish, or preparation of the manuscript.*<br>• **YES** - Specify the role(s) played.<br><br>* typeset	
**Competing Interests**<br><br>Use the instructions below to enter a competing interest statement for this submission. On behalf of all authors, disclose any competing interests that could be perceived to bias this work—acknowledging all financial support and any other relevant financial or non-financial competing interests.<br><br>This statement is required for submission and **will appear in the published article** if the submission is accepted. Please make sure it is accurate and that any funding sources listed in your Funding Information later in the submission form are also declared in your Financial Disclosure	The authors have declared that no competing interests exist.

Powered by Editorial Manager® and ProduXion Manager® from Aries Systems Corporation

statement.<br><br>View published research articles from *PLOS ONE* for specific examples.<br><br>**NO authors have competing interests**<br><br>Enter: *The authors have declared that no competing interests exist.*<br><br>**Authors with competing interests**<br><br>Enter competing interest details beginning with this statement:<br><br>*I have read the journal's policy and the authors of this manuscript have the following competing interests: [insert competing interests here]*<br><br>* typeset	
**Ethics Statement**<br><br>Enter an ethics statement for this submission. This statement is required if the study involved:<br><br>• Human participants<br>• Human specimens or tissue<br>• Vertebrate animals or cephalopods<br>• Vertebrate embryos or tissues<br>• Field research<br><br>Write "N/A" if the submission does not require an ethics statement.<br><br>General guidance is provided below. Consult the submission guidelines for detailed instructions. **Make sure that all information entered here is included in the Methods section of the manuscript.**	N/A

Powered by Editorial Manager® and ProduXion Manager® from Aries Systems Corporation

**Format for specific study types**<br><br>**Human Subject Research (involving human participants and/or tissue)**<br>• Give the name of the institutional review board or ethics committee that approved the study<br>• Include the approval number and/or a statement indicating approval of this research<br>• Indicate the form of consent obtained (written/oral) or the reason that consent was not obtained (e.g. the data were analyzed anonymously)<br><br>**Animal Research (involving vertebrate animals, embryos or tissues)**<br>• Provide the name of the Institutional Animal Care and Use Committee (IACUC) or other relevant ethics board that reviewed the study protocol, and indicate whether they approved this research or granted a formal waiver of ethical approval<br>• Include an approval number if one was obtained<br>• If the study involved *non-human primates*, add *additional details* about animal welfare and steps taken to ameliorate suffering<br>• If anesthesia, euthanasia, or any kind of animal sacrifice is part of the study, include briefly which substances and/or methods were applied<br><br>**Field Research**<br><br>Include the following details if this study involves the collection of plant, animal, or other materials from a natural setting:<br>• Field permit number<br>• Name of the institution or relevant body that granted permission	
**Data Availability**<br><br>Authors are required to make all data underlying the findings described fully available, without restriction, and from the time of publication. PLOS allows rare exceptions to address legal and ethical concerns. See the PLOS Data Policy and FAQ for detailed information.	Yes - all data are fully available without restriction

Powered by Editorial Manager® and ProduXion Manager® from Aries Systems Corporation

A Data Availability Statement describing where the data can be found is required at submission. Your answers to this question constitute the Data Availability Statement and **will be published in the article**, if accepted.<br><br>**Important:** Stating 'data available on request from the author' is not sufficient. If your data are only available upon request, select 'No' for the first question and explain your exceptional situation in the text box.<br><br>Do the authors confirm that all data underlying the findings described in their manuscript are fully available without restriction?	
**Describe where the data may be found in full sentences. If you are copying our sample text, replace any instances of XXX with the appropriate details.**<br><br>• If the data are **held or will be held in a public repository**, include URLs, accession numbers or DOIs. If this information will only be available after acceptance, indicate this by ticking the box below. For example: *All XXX files are available from the XXX database (accession number(s) XXX, XXX.)*.<br>• If the data are all contained **within the manuscript and/or Supporting Information files**, enter the following: *All relevant data are within the manuscript and its Supporting Information files.*<br>• If neither of these applies but you are able to provide **details of access elsewhere**, with or without limitations, please do so. For example:<br><br>*Data cannot be shared publicly because of [XXX]. Data are available from the XXX Institutional Data Access / Ethics Committee (contact via XXX) for researchers who meet the criteria for access to confidential data.*<br><br>*The data underlying the results presented in the study are available from (include the name of the third party*	All relevant data are within the manuscript and its Supporting Information files.

and contact information or URL).<br>• This text is appropriate if the data are owned by a third party and authors do not have permission to share the data.<br><br>* typeset	
Additional data availability information:	

Manuscript Click here to access/download;Manuscript;Manuscipt.docx

Research on the Collaborative Agglomeration Level and Spatial Correlation of Intercity Manufacturing Industry: An Empirical Study Based on the Central Cities of the Yangtze River Delta

ZHU Kai [1, 2], SUN Wanxiang [1]

(1.School of Design and Architecture, Zhejiang University of Technology, Hangzhou 310023, Zhejiang, China; 2. School of Architecture, Southeast University, Nanjing 210096, Jiangsu, China)

Abstract: Taking 27 cities in the central area of the Yangtze River Delta as an example, the time section data from 2009 to 2019 are selected, and the location entropy index and the modified E-G index are introduced to measure the collaborative agglomeration level of intercity manufacturing industry in the Yangtze River Delta. The spatial weight matrix is constructed based on the highway mileage between cities. Using Moran’s index and Local Moran’s index, this article analyzes the spatial correlation of the collaborative agglomeration level of intercity manufacturing industry in the Yangtze River Delta. The results show that: (1) The overall manufacturing industry agglomeration degree of cities in the central area of the Yangtze River Delta shows a fluctuating downward trend. The manufacturing industry agglomeration degree of Jiangsu and Zhejiang Province has decreased, that of most cities in Anhui Province has increased steadily. (2) The level of manufacturing industry collaborative agglomeration between Shanghai, Nanjing, Hangzhou and Jiangsu and Zhejiang cities showed a downward trend, while the level of collaborative agglomeration with Anhui cities increased steadily. The level of manufacturing industry collaborative agglomeration between Wuxi, Suzhou, Nanjing and cities with obvious advantages in manufacturing industry agglomeration degree shows an upward trend, while the level of collaborative agglomeration with other cities has declined. The collaborative agglomeration level of intercity manufacturing industry in Hefei has generally improved, especially the quality and depth of manufacturing industry collaborative agglomeration between Hefei and Anhui cities have significantly improved. (3) The regions with high level of intercity manufacturing industry collaborative agglomeration in the Yangtze River Delta are still mainly concentrated in Jiangsu and Zhejiang provinces. Although there has been a trend of westward transfer in the past decade, the overall change range is small, and the spatial correlation of the level of intercity manufacturing industry collaborative agglomeration shows a weakening trend as a whole, and the spatial differences continue to narrow.

Keywords: intercity; manufacturing; collaborative agglomeration; spatial correlation; Yangtze River Delta

1. Introduction

1.1. Background

Building a new development pattern of mutual promotion between domestic and international dual circulation is a major judgment on China's current economic situation and policies, among which internal circulation is an important focus for promoting high-quality development of China's dual circulation. As an important part of internal circulation, the circulation in the manufacturing industry has become the foundation of urban and even regional economic development with the continuous promotion of re industrialization and the return of the manufacturing industry. At the same time, promoting its high-quality development is also an inherent requirement for building a modern economic system. With the successive implementation of a new round of national and regional development strategies, the free circulation and optimized allocation of various production factors across the country and even globally have significantly accelerated. The industrial connections between different regions are becoming increasingly close, and the aggregation effect of capital is gradually emerging. Integration, especially industrial integration, has become a top priority for regional future development.

The Yangtze River Delta, as the leading force driving China's economic development and reform and opening up, is an important model area for regional integration development and the main battlefield for promoting high-quality development of the manufacturing industry. With the deepening of supply side structural reform, the high-quality integrated development of the Yangtze River Delta is gradually shifting towards a pattern of coordinated, integrated, and balanced development. In the future, the development of regional intercity industries will shift from competition to cooperation, and from siphon to interaction. Comprehensively promoting the transformation and upgrading of the manufacturing industry, actively cultivating new driving forces and engines, striving to create characteristic and advantageous industries, and laying a collaborative development pattern of interactive and complementary regional industrial chains are not only urgent need to optimize the internal industrial structure of the Yangtze River Delta urban agglomeration, but also inevitable stage for the integration of the Yangtze River Delta region to move towards higher levels and higher quality [1]. In this context, as the core content of regional integration, the collaborative agglomeration of intercity manufacturing industry is both a trend and an inevitable requirement. Measuring the collaborative agglomeration level of intercity manufacturing industry in the process of regional integration in the Yangtze River Delta is of great practical significance for clarifying the development direction of the manufacturing industry and promoting high-quality development of the integration of the Yangtze River Delta.

1.2. Literature Review

The concept of industrial collaborative agglomeration originated from abroad, and Ellison & Glaeser defined the spatial agglomeration phenomenon of industries as "co-agglomeration" [2]. Since then, many scholars have continuously enriched the research on industrial collaborative agglomeration and achieved fruitful theoretical results. Marshall's externality theory has laid the foundation for the emergence of the theory of industrial synergy and agglomeration [3]. The "center-periphery" model

constructed by Krugman & Paul incorporates spatial factors into relevant research, and further develops the relevant content of industrial collaborative agglomeration [4]. Venables constructed a vertical correlation model from the perspective of heterogeneity, and pointed out that upstream and downstream industries with forward and backward connections tend to be closer to each other in space [5]. Ellison et al. explored the degree of collaborative agglomeration between different industries in the United States based on relevant data analysis, and validated three influencing factors proposed by Marshall: the connection between intermediate inputs and final product suppliers, shared labor markets, increased information exchange and innovation opportunities [6]. King et al. introduced tax competition into the CPVL model and found that tax and transportation costs have the same impact on industrial agglomeration [7]. Forslid and Midelfart introduced the CPVL model to optimize the collaborative agglomeration of upstream and downstream industries in countries where government departments receive high wage openness [8]. Suedekum introduced the housing product sector into the Krugman center periphery model to analyze the industrial agglomeration characteristics of the three sectors [9]. When Kolko and Neumark analyzed the phenomenon of internal agglomeration in the service industry, they found that the internal agglomeration in the service industry was generated through trade exchanges between industries and the Spillover effect of knowledge [10]. Koh and Riedel replaced vertical correlation with horizontal correlation and established a three sector model for the two countries. They also found that there is spatial synergistic agglomeration between the intermediate product industry and the final product industry [11].

At present, research on industrial collaborative agglomeration mainly focuses on the collaborative agglomeration between manufacturing industry and productive service industry, covering multiple aspects such as existence analysis, collaborative agglomeration effect analysis, and collaborative agglomeration mechanism analysis. In terms of existence analysis, Krugman found that a large number of manufacturing industries in the United States have a high degree of production agglomeration, which is not limited to high-tech industries [12]. Duranto & Overman took micro enterprises as the research object, and adopted the method of econometric analysis to analyze the existence of collaborative agglomeration between productive service industry and manufacturing industry. The research results show that Marshall externality, knowledge spillovers and direct trade relations, circular cumulative effects and policy intervention are the main reasons for industrial collaborative agglomeration [13]. In terms of collaborative agglomeration effect analysis, Lu Jianbao conducted a study on the synergistic effect of productive service industry based on manufacturing industry agglomeration, and measured the impact of manufacturing industry agglomeration on productive service industry agglomeration. He found that manufacturing industry has a agglomeration driving effect on productive service industry, and manufacturing industry agglomeration is the main factor leading to the agglomeration of productive service industry [14]. Sheng Feng discussed the impact of the spatial agglomeration of productive service industry on the upgrading of manufacturing industry and its spatial spillover effect. He found that the spatial agglomeration of productive service industry

can drive the upgrading of manufacturing industry [15]. In addition, some scholars have also proposed that the collaborative agglomeration between manufacturing industry and productive service industry has a significant positive promoting effect on industrial structure upgrading [16], regional and urban economic growth [17], and the improvement of total factor productivity [18]. In terms of collaborative agglomeration mechanism analysis, Gallagher subdivided transportation costs into physical and information transportation costs when studying the formation mechanism of industrial collaborative agglomeration, and analyzed that the Marshall factor under heterogeneous transportation costs is the source of the formation of industrial collaborative agglomeration [19]. Gabe & Abel examined the key role of Marshall's third factor and found that professional labor with similar knowledge tend to collaborate more, and the importance of knowledge sharing caused by this collaboration was greater at the urban scale than at the state level [20]. Through theoretical model deduction, Yang Renfa proposed that the effect of industrial collaborative agglomeration on environmental pollution is achieved through enterprise scale, technological progress, and economic structure [21]. Zhang Zhidong and Qin Shuyue believed that the role of industrial agglomeration in green development is achieved through the dual effects of scale effect and crowding effect [22]. Chen Xi et al. proposed that institutions and policies, information transmission capabilities, economic development levels, manufacturing industry labor supply and transportation facility level are the main factors affecting the regional differences in collaborative agglomeration among manufacturing industry in China [23].

Overall, there are still some shortcomings in existing research, mainly including three aspects. Firstly, in terms of research perspective, industrial collaborative agglomeration refers to the agglomeration of different industries in a specific space, with a dual attribute of "industry-space". However, some studies have focused more on the single attribute of industry and explored the phenomenon of industrial collaborative agglomeration from an economic perspective, and neglected the importance of spatial attributes [24,25,26]. Secondly, in terms of research objects, current research on the measurement of collaborative agglomeration level is mostly based on regions or provinces, and research focusing on the measurement of intercity manufacturing industry collaborative agglomeration level is still rare [27,28,[29]. Thirdly, in terms of research content, existing research has mostly focused on the collaborative agglomeration between manufacturing industry and productive service industry or other industries, and there is relatively little research on the collaborative agglomeration between individual industries in the manufacturing industry [30,31]. However, as an important carrier of regional integration in the Yangtze River Delta, the collaborative agglomeration of manufacturing industry between cities is also a focus of integration attention. Measuring the level of intercity manufacturing industry collaborative agglomeration will help better promote the process of regional integration in the Yangtze River Delta.

2. Objects and Methods

2.1. Research Objects and Research Ideas

2.1.1. Research Objects

In the “Outline of the Yangtze River Delta Regional Integration Development Plan” issued by the State Council in 2019, 27 cities including Shanghai, Nanjing, Wuxi, Changzhou, Suzhou, Nantong, Yangzhou, Zhenjiang, Yancheng, Taizhou, Hangzhou, Ningbo, Wenzhou, Huzhou, Jiaxing, Shaoxing, Jinhua, Zhoushan, Taizhou, Hefei, Wuhu, Ma’anshan, Tongling, Anqing, Chuzhou, Chizhou, Xuancheng are defined as the central cities of the Yangtze River Delta. The manufacturing industry in the central cities of the Yangtze River Delta has a strong foundation, which can radiate and drive the high-quality development of the manufacturing industry in the entire Yangtze River Delta region. It is a leading and demonstration area for integrated development, and measuring the level of intercity manufacturing industry collaborative agglomeration has certain typicality and representation. This article selects the central cities of the Yangtze River Delta as the research objects and focuses on the manufacturing industry. The sample study spans from 2009 to 2019, with data mainly sourced from statistical yearbooks and statistical bureau websites of various provinces and cities, as well as China’s urban statistical yearbooks.

2.1.2. Research Ideas

Building an integrated factor market is the key to regional integration. Generally speaking, a large economic volume brings about the agglomeration of production factors, which in turn further leads to the coordinated development of industries. With the deepening of integration, the flow and allocation of various production factors between regions are constantly accelerating, breaking the inherent understanding that economic volume determines factor agglomeration. The relationship between cities is becoming increasingly close, and the coordinated development of industries is no longer only influenced by urban economic volume, but also by the relationship between cities. The main idea of this research is to measure the agglomeration level of manufacturing industry and the level of collaboration based on agglomeration level, and then analyze the spatial synergy relationship of collaboration level.

Starting from the fact of agglomeration in the intercity manufacturing industry of the Yangtze River Delta, this article uses the industrial agglomeration index to measure the level of manufacturing industry agglomeration in 27 cities in the central area of the Yangtze River Delta to analyzes the differences in manufacturing industry agglomeration among different cities, and then uses the industrial collaborative agglomeration index to measure whether the manufacturing industry agglomeration levels of different cities are coordinated. Collaborative agglomeration of manufacturing industry is an advanced stage of manufacturing industry agglomeration, which not only reflects the close technological and economic connections between different cities, but also serves as an important basis for predicting the future development of intercity manufacturing industry in the Yangtze River Delta. In order to further explore the relationship between collaborative agglomeration and spatial distance of the intercity manufacturing industry in the Yangtze River Delta, that is, whether manufacturing industry collaborative agglomeration is short-distance or long-distance collaboration, this article uses Moran’s I and Local Moran’s I to conduct spatial correlation analysis on the level of intercity manufacturing industry

collaborative agglomeration in the Yangtze River Delta to test whether the level of intercity manufacturing industry collaborative agglomeration is influenced by the proximity of urban spatial distances, and to provide more scientific and practical guidance for the development direction of the manufacturing industry in the Yangtze River Delta.

2.2. Research Methods and Indicator Selection

2.2.1. Measurement of Collaborative Agglomeration Level

(1) Industrial Agglomeration Index

Location entropy is an indicator that measures the degree of specialization of a specific industry in a certain region within the entire region. It is usually used to represent the balanced development of industries between cities or the level of industrial agglomeration. It can eliminate the impact of regional scale differences and more accurately reflect the spatial distribution of industrial agglomeration. Therefore, this article uses the location entropy index to calculate the manufacturing industry agglomeration level of central cities in the Yangtze River Delta. The specific calculation formula is as follows:

$$\mathrm{LQ}_{\mathrm{kj}} = \frac{q_{kj}/q_j}{q_k/q} \quad (1)$$

where $\mathrm{LQ}_{\mathrm{kj}}$ and q_{kj} respectively represent the location entropy and the relevant indicators of the k industry in j city; q_j represents the relevant indicators of all industries in j city; q_k represents the relevant indicators of the k industry in the entire region; and q represents the relevant indicators of all industries in the entire region. In order to better reflect the degree of industrial agglomeration and eliminate the impact of economic development level and price difference of the year, this article uses the number of urban unit employees at the end of the period as the main indicator to measure industrial agglomeration. Then, q_{kj} represents the number of urban unit employees in the manufacturing industry of j city at the end of the period; q_j represents the number of urban unit employees in all industries of j city at the end of the period; q_k represents the number of urban unit employees in the manufacturing industry within the entire region at the end of the period; and q represents the number of urban unit employees in all industries within the entire region at the end of the period. The larger the value of $\mathrm{LQ}_{\mathrm{kj}}$, the higher the level of manufacturing industry agglomeration in the city. Generally speaking, when $\mathrm{LQ}_{\mathrm{kj}}>1$, it indicates that the k industry in j city has advantages throughout the entire region, which can be considered that the k industry is clustered in j city. When $\mathrm{LQ}_{\mathrm{kj}}<1$, it indicates that the k industry in j city has disadvantages throughout the entire region.

(2) Industrial Collaborative Agglomeration Index

The E-G index constructed by Ellison & Glaeser measures the strength of the relationship between enterprises' "co-location" according to the relevance of

enterprise location, and weed out the market concentration ratio to eliminate the impact of enterprise size differences on industrial agglomeration, so as to more accurately measure the agglomeration degree of industrial geographical distribution [2]. To solve the problem of data acquisition and better adapt to the development of China's manufacturing industry, this article uses the revised E-G index to calculate the level of collaborative agglomeration of the intercity manufacturing industry in the Yangtze River Delta through the relative differences in industrial agglomeration. The calculation formula is as follows:

$$\mathrm{CO} = 1 - \left|\mathrm{LQ_{ki}} - \mathrm{LQ_{kj}}\right| / (\mathrm{LQ_{ki}} + \mathrm{LQ_{kj}}) \tag{2}$$

where $\mathrm{LQ_{ki}}$ and $\mathrm{LQ_{kj}}$ respectively represent the agglomeration level of k industry in i city and j city, which are generally measured by location entropy.

This formula mainly reflects the quality of industrial collaborative agglomeration, without considering the depth of industrial collaborative agglomeration. Therefore, referring to the research of Chen Jianjun et al. [32] and Zhang Hu et al. [33], the above formula is further improved by considering both the quality and depth of industrial collaborative agglomeration, in order to better reflect the overall level of collaborative agglomeration of the intercity manufacturing industry in the Yangtze River Delta. The calculation formula is as follows:

$$\mathrm{COO} = 1 - \left|\mathrm{LQ_{ki}} - \mathrm{LQ_{kj}}\right| / (\mathrm{LQ_{ki}} + \mathrm{LQ_{kj}}) + \left|\mathrm{LQ_{ki}} + \mathrm{LQ_{kj}}\right| \tag{3}$$

2.2.2. Spatial Correlation Analysis

(1) Spatial Weight Matrix

The establishment of spatial weight matrix is the foundation for conducting regional spatial correlation analysis and an important tool for reflecting regional spatial relationships [34,35]. Commonly used spatial weight matrices include adjacent spatial weight matrix, geographical metric space weight matrix, economic metric space weight matrix, etc. The geographical metric space weight matrix regards geographical distance as an important factor in the measurement of interaction effect between space units, that is, a specific spatial unit will not only have interactive effects on its surrounding spatial units, but also will have interactive effects on non adjacent spatial units. The strength of this interactive influence is influenced by the geographical distance between spatial units. The geographical metric space weight matrix can more comprehensively and actually reflect the interaction effect between spatial units. Therefore, this article selects the reciprocal of the distance between cities in the central area of the Yangtze River Delta as the constituent element of the spatial matrix, and its specific expression is as follows:

$$W_{ij} = \begin{cases} \frac{1}{d_{ij}} & i \neq j \\ 0 & i = j \end{cases} \tag{4}$$

To ensure the consistency of calculation standards and the availability of data, this article uses the mileage of highways between cities as the measure of spatial distance between cities. The value of d_{ij} is the mileage of highways between cities.

The larger the mileage of highways between cities, the smaller the weight, and vice versa.

(2) Measurement of Spatial Correlation

Spatial autocorrelation is an important basis for testing whether a certain attribute in different geographical regions has spatial correlation features [36]. There are two main methods to measure the spatial correlation of variables: Moran’s I and Local Moran’s I. Among them, Moran’s I is mainly used to reflect the spatial correlation characteristics of all spatial units, while Local Moran’s I can reflect the characteristics of spatial heterogeneity between spatial units. The specific calculation formulas are as follows:

$$Moran's\ I=\frac{\sum_{i=1}^{n}\sum_{j=1}^{n}w_{ij}(x_i-\bar{x})(x_j-\bar{x})}{S^2\sum_{i=1}^{n}\sum_{j=1}^{n}w_{ij}} \tag{5}$$

$$Local\ Moran's\ I=\frac{(x_i-\bar{x})}{S^2}\sum_{j=1}^{n}w_{ij}(x_i-\bar{x}) \tag{6}$$

where n is the number of cities; x_i and x_j respectively represent the observations of i city and j city; w_{ij} represents spatial weight matrix; $S^2=\frac{\sum_{i=1}^{n}(x_i-x)^2}{n}$ is the sample variance; and $\sum_{i=1}^{n}\sum_{j=1}^{n}w_{ij}$ is the sum of all spatial weights. The Moran’s I has values between [-1,1]. When Moran’s I>0, it indicates the existence of spatial positive correlation; when Moran’s I<0, it indicates the existence of spatial negative correlation; and when Moran's I=0, it indicates the absence of spatial correlation. The closer the value of Moran’s I is to 1, the stronger the spatial positive correlation of the collaborative agglomeration level of intercity manufacturing industry. The closer the value of Moran’s I is to -1, the stronger the spatial negative correlation of the collaborative agglomeration level of intercity manufacturing industry. The values of the Local Moran’s I are also between [-1,1]. When Local Moran’s I>0, it indicates that the observed values between two cities exhibit a co-directional correlation of “high -high” or “low-low” clustering. When Local Moran’s I<0, it indicates that the observed values between two cities exhibit a reverse correlation of “high-low” or “low-high” clustering. When Local Moran’s I=0, Indicates that there is no spatial correlation between the observed values between two cities. The closer the absolute value of Local Moran’s I is to 1, the more obvious the spatial correlation characteristics between cities.

3. Measurement of the Collaborative Agglomeration Level of Manufacturing Industry between Cities in the Yangtze River Delta

3.1. Agglomeration Level of Manufacturing Industry

Using the the number of urban unit employees at the end of the period as the main indicator, the agglomeration level of manufacturing industry in the central cities of the Yangtze River Delta was measured, and the results are shown in Table 1. From the average level of manufacturing industry agglomeration in various cities, the agglomeration degree of manufacturing industry in Suzhou, Jiaxing, Wuxi, Changzhou, and Ningbo all exceeds 4.00, which is much higher than other cities. The

development of manufacturing industry in these cities started early, with a relatively strong foundation, and the agglomeration of manufacturing industry has formed a certain scale. The agglomeration degree of manufacturing industry in Jinhua, Zhoushan, Anqing, and Chizhou is still around 1, which is far lower than the average level of central cities in the Yangtze River Delta. The analysis may be related to their urban development level and leading industries, and their manufacturing industry foundation is relatively weak and has not yet formed a agglomeration scale.

From the perspective of temporal changes, the overall manufacturing industry agglomeration degree of central cities in the Yangtze River Delta shows a fluctuating downward trend, which may be related to the transformation and upgrading of their industrial structure and the development towards service-oriented direction. Among the seven core cities in the central area of the Yangtze River Delta, namely Shanghai, Nanjing, Wuxi, Suzhou, Hangzhou, Ningbo, and Hefei, the manufacturing industry agglomeration of Shanghai, Nanjing, and Hangzhou has shown an overall downward trend, which may be related to the relocation of their manufacturing industry and the transformation towards service industry development. Suzhou, Wuxi, and Ningbo have shown an overall upward trend in manufacturing agglomeration in the past decade due to their strong manufacturing industry foundation, and the process of manufacturing industry upgrading is constantly advancing. Due to the continuous influx of manufacturing industry in recent years, the agglomeration degree of manufacturing industry in Hefei has begun to show an upward trend. Looking at the overall changes in various provinces and cities, the agglomeration degree of manufacturing industry in most cities in Jiangsu and Zhejiang provinces has decreased, while the agglomeration degree of manufacturing industry in most cities in Anhui province has steadily increased, and the phenomenon of manufacturing industry shifting westward is obvious (Figure 1).

Table 1. The Manufacturing Industry Agglomeration Degree of Central Cities in the Yangtze River Delta.

Region/City	2009	2010	2011	2012	2013	2014	2015	2016	2017	2018	2019	Average
Yangtze River Delta	3.90	3.89	3.88	3.30	2.60	2.58	2.68	2.84	3.00	3.18	3.38	3.20
Shanghai	3.48	3.43	3.65	3.43	2.49	2.21	2.26	2.44	2.49	2.62	2.31	2.80
Nanjing	3.54	3.60	3.55	3.16	1.97	1.82	1.98	2.00	2.03	2.11	2.29	2.55
Wuxi	5.10	5.22	5.22	4.68	4.17	4.30	4.49	4.67	5.11	5.61	5.84	4.95
Changzhou	4.33	4.22	3.98	3.99	3.28	3.39	3.79	4.05	4.39	4.64	5.21	4.12
Suzhou	6.48	6.56	5.94	5.75	4.94	5.22	5.51	5.89	6.28	6.92	7.20	6.06
Nantong	4.55	4.57	4.32	3.96	1.58	1.58	1.78	1.92	1.87	1.95	2.32	2.76
Yangzhou	3.24	3.28	3.36	3.14	1.83	2.05	2.11	2.22	2.28	2.33	2.23	2.55
Zhenjiang	4.55	4.70	4.44	4.23	3.38	3.75	3.95	4.22	3.91	3.84	3.70	4.06
Yancheng	2.90	3.01	3.00	2.74	2.22	2.24	2.23	2.33	2.32	2.18	2.48	2.51
Taizhou	3.84	3.93	3.85	3.46	2.12	2.06	2.20	2.23	2.34	2.02	2.36	2.76

Hangzhou	3.34	3.11	3.37	2.48	1.87	1.81	1.87	1.90	2.10	2.30	2.40	2.41
Ningbo	4.28	4.33	4.87	4.09	3.34	3.34	3.46	3.57	3.81	4.42	4.67	4.02
Wenzhou	4.53	4.48	4.02	3.30	2.42	2.35	2.29	2.47	2.34	2.39	2.92	3.05
Huzhou	4.82	4.82	4.20	3.37	2.76	2.88	2.97	3.22	3.46	3.74	4.15	3.67
Jiaxing	6.24	6.19	5.95	5.38	4.45	4.52	4.55	4.80	5.10	5.73	6.34	5.39
Shaoxing	3.74	3.34	2.92	2.43	2.03	2.00	1.99	2.03	2.30	2.31	2.82	2.54
Jinhua	2.59	2.80	2.71	1.57	1.28	1.23	1.28	1.31	1.61	1.56	2.09	1.82
Zhoushan	2.58	2.50	2.25	2.00	1.66	1.69	1.75	1.84	1.59	1.75	2.05	1.97
Taizhou	3.03	3.13	3.50	3.00	2.58	2.50	2.40	2.79	2.97	3.37	3.99	3.02
Hefei	2.20	2.39	2.57	2.11	1.71	1.88	1.95	2.03	2.15	2.05	2.21	2.11
Wuhu	3.76	4.05	3.50	3.38	2.90	2.94	3.11	3.41	3.53	4.03	4.15	3.52
Ma'anshan	4.32	4.29	3.48	3.08	2.13	2.20	2.31	2.42	2.51	3.29	3.42	3.04
Tongling	4.13	4.09	4.53	4.03	3.05	3.09	2.86	3.04	3.24	3.45	3.87	3.58
Anqin	1.22	1.22	1.28	1.10	1.78	1.81	2.13	2.30	2.40	2.99	2.92	1.92
Chuzhou	1.81	1.70	1.79	1.75	2.13	2.13	2.48	2.69	3.22	3.55	4.15	2.49
Chizhou	1.31	1.41	1.43	1.23	1.34	1.51	1.55	1.76	1.96	2.35	2.34	1.65
Xuancheng	2.35	2.47	5.70	1.38	1.20	2.22	2.42	2.59	2.82	3.54	3.64	2.76

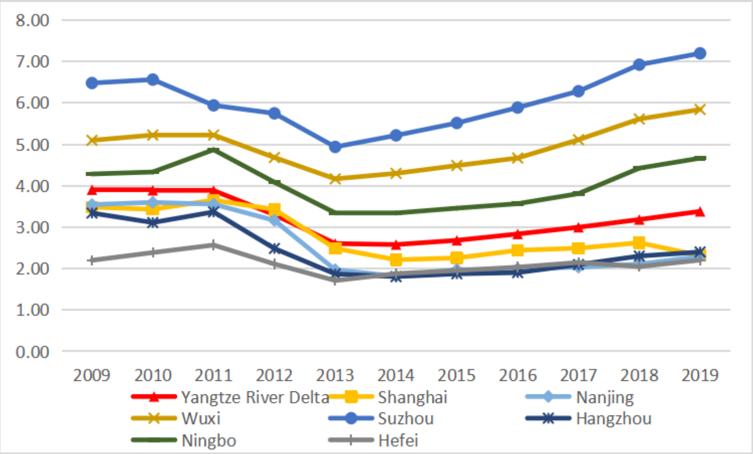

Figure 1. Changes in Manufacturing Industry Agglomeration of ~~Central Cities and~~ Core Cities in the Yangtze River Delta From 2009 to 2019.

3.2. Collaboration Level of Intercity Manufacturing Industry

On the basis of measuring the agglomeration level of manufacturing industry in various cities, in order to further explore whether the agglomeration level of manufacturing industry among cities is coordinated, this article uses formula (3) to calculate the collaboration level of intercity manufacturing industry in the Yangtze River Delta. With the help of ArcGIS software, 27 cities are regarded as independent point elements, and the line between points represents the collaboration level of intercity manufacturing industry. The collaboration level is represented by the depth of the color. The darker the color of the line, the higher the collaboration level of intercity manufacturing industry, and vice versa. Figure 2 shows the calculation results of the collaboration level of intercity manufacturing industry in the Yangtze River Delta in 2009, 2012, 2015, and 2019.

From the graph, it can be observed that the collaboration level of intercity

manufacturing industry between cities such as Suzhou, Jiaxing, Wuxi, Ningbo, and other cities is relatively high, while the collaboration level of intercity manufacturing industry between cities in Anhui Province, northern Jiangsu, southern Zhejiang, and other cities is relatively low, showing an overall trend of weakening from the center to the periphery. In terms of specific measurement values, the average collaboration level of intercity manufacturing industry between cities such as Suzhou-Jiaxing, Wuxi-Suzhou, Wuxi-Jiaxing, Suzhou-Changzhou, Suzhou-Zhenjiang, Suzhou-Ningbo, Suzhou-Huzhou, Suzhou-Tongling, Suzhou-Wuhu, Ningbo-Jiaxing, etc. all exceeds 10.00. Both the quality and depth of collaborative agglomeration in the manufacturing industry have obvious advantages. The analysis found that these cities have a relatively high degree of manufacturing industry agglomeration and small intercity differences, resulting in a relatively high collaboration level. The collaboration level of intercity manufacturing industry between cities such as Yangzhou-Chizhou, Yancheng-Chizhou, Hangzhou-Chizhou, Hangzhou-Xuancheng, Shaoxing-Chizhou, Jinhua-Zhoushan, Hefei-Anqing, and Hefei-Chizhou is still below 5.00. The analysis may be due to the presence of cities with relatively low manufacturing industry agglomeration, resulting in significant differences in manufacturing industry agglomeration between cities. Therefore, there is significant room for improvement in the quality and depth of collaborative agglomeration of manufacturing industry among these cities.

From the perspective of temporal changes, the trend of manufacturing industry transfer from Jiangsu and Zhejiang provinces to Anhui province is obvious, and the collaborative agglomeration degree of intercity manufacturing industry between cities in Anhui province is constantly strengthening. In terms of specific cities, the collaborative agglomeration level of intercity manufacturing industry between Shanghai, Nanjing, Hangzhou and cities in Jiangsu and Zhejiang provinces has shown a downward trend in the past decade, while the collaborative agglomeration level of intercity manufacturing industry between cities in Anhui province has steadily improved. The collaborative agglomeration level of intercity manufacturing industry between Wuxi, Suzhou, Ningbo and cities with obvious advantages in manufacturing industry agglomeration has shown an upward trend in the past decade, while the collaborative agglomeration level of intercity manufacturing industry with other cities has also decreased. The collaborative agglomeration level of intercity manufacturing industry in Hefei has generally improved in the past decade, especially with the significant improvement in the collaborative agglomeration quality and depth of intercity manufacturing industry with various cities in Anhui Province. Overall, the regions with high collaborative agglomeration level of intercity manufacturing industry in the Yangtze River Delta are still mainly concentrated in Jiangsu and Zhejiang provinces. Although there has been a trend of westward transfer in the past decade, the overall change is not significant. It can be found that the trend of the collaborative agglomeration level of intercity manufacturing industry is related to the trend of manufacturing industry agglomeration in each city itself. Generally speaking, the collaborative agglomeration level of intercity manufacturing industry between cities with high manufacturing industry agglomeration is also relatively high, and the

collaborative agglomeration level of intercity manufacturing industry rises and falls with the rise and fall of manufacturing industry agglomeration.

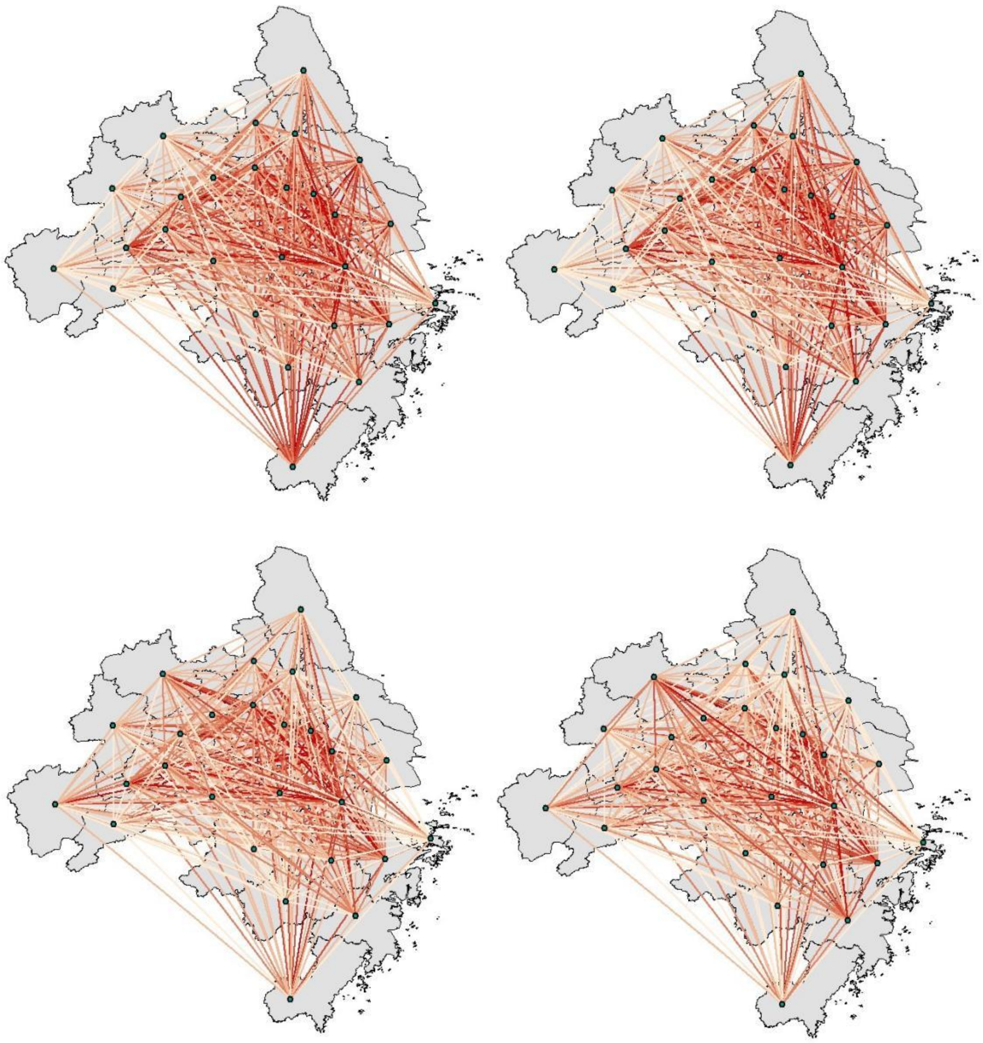

Figure 2. Collaborative Agglomeration Level of Intercity Manufacturing Industry in the Yangtze River Delta in 2009, 2012, 2015 and 2019.

4. Spatial Correlation Analysis of the Collaborative Agglomeration Level of Manufacturing Industry between Cities in the Yangtze River Delta

From the above calculation results, it can be seen that the collaborative agglomeration level of intercity manufacturing industry in the Yangtze River Delta shows a non equilibrium feature, with both short distance and long distance collaborative phenomena occurring simultaneously. To explore whether the collaborative agglomeration characteristics of manufacturing industry between different cities have spatial dependence, this article uses the Moran's I and Local Moran's I indices to conduct spatial correlation analysis on the collaborative

agglomeration level of intercity manufacturing industry in the Yangtze River Delta, in order to test the mutual relationship between the collaborative agglomeration level of intercity manufacturing industry and spatial distance.

4.1. Spatial Autocorrelation Test

In order to test the overall spatial correlation of collaborative agglomeration level of intercity manufacturing industry in the Yangtze River Delta, according to the above measured collaborative agglomeration level of intercity manufacturing industry, the overall collaborative agglomeration level in each city is expressed by summing up the values between each city and 26 other cities. Then, Stata software is used to calculate the Moran's I of the collaborative agglomeration level of intercity manufacturing industry in the Yangtze River Delta after standardizing the spatial weight matrix by using the geographical metric space weight matrix. The calculation results are shown in Table 3.

Table 3. Moran' I Index of Collaborative Agglomeration Level of Intercity Manufacturing Industry in the Yangtze River Delta from 2009 to 2019.

Year	Moran's I	Sd（I）	Z value	P value
2009	0.083	0.033	3.719	0.000
2010	0.074	0.032	3.468	0.000
2011	0.041	0.033	2.441	0.007
2012	0.070	0.033	3.318	0.000
2013	0.025	0.032	1.970	0.024
2014	0.021	0.032	1.859	0.032
2015	0.030	0.032	2.148	0.016
2016	0.025	0.032	1.990	0.023
2017	0.025	0.032	1.990	0.023
2018	0.013	0.032	1.593	0.056
2019	0.009	0.032	1.469	0.071

As shown in the table, the Moran's I distribution of the collaborative agglomeration level of intercity manufacturing industry in the Yangtze River Delta from 2009 to 2019 ranges from 0.01 to 0.08, with values greater than 0, indicating a spatial positive correlation between the collaborative agglomeration level of intercity manufacturing industry. In terms of significance, the p-values of the collaborative agglomeration level of intercity manufacturing industry from 2009 to 2019 were all less than 0.1, passing the 10% significance test, indicating that there is a significant spatial positive correlation in the collaborative agglomeration level of intercity manufacturing industry in the Yangtze River Delta, reflecting significant spatial agglomeration characteristics. From the perspective of temporal changes, the overall spatial correlation shows a weakening trend, indicating that the impact of spatial distance on the collaborative agglomeration of intercity manufacturing industry is continuously decreasing, and the cross city collaborative cooperation of manufacturing industry is gradually breaking through the limitations of spatial distance.

4.2. Local Spatial Correlation Analysis

Due to the inability of the Moran's I index to reflect the spatial clustering characteristics and spatial dependence of the collaborative agglomeration level of intercity manufacturing industry, this article further studies the local spatial correlation of the collaborative agglomeration level of intercity manufacturing industry in the Yangtze River Delta by drawing a Moran scatter plot. This article selects Moran scatter plots for comparative analysis of the collaborative agglomeration level of intercity manufacturing industry in four years of 2009, 2012, 2015, and 2019, as shown in Figure 3.

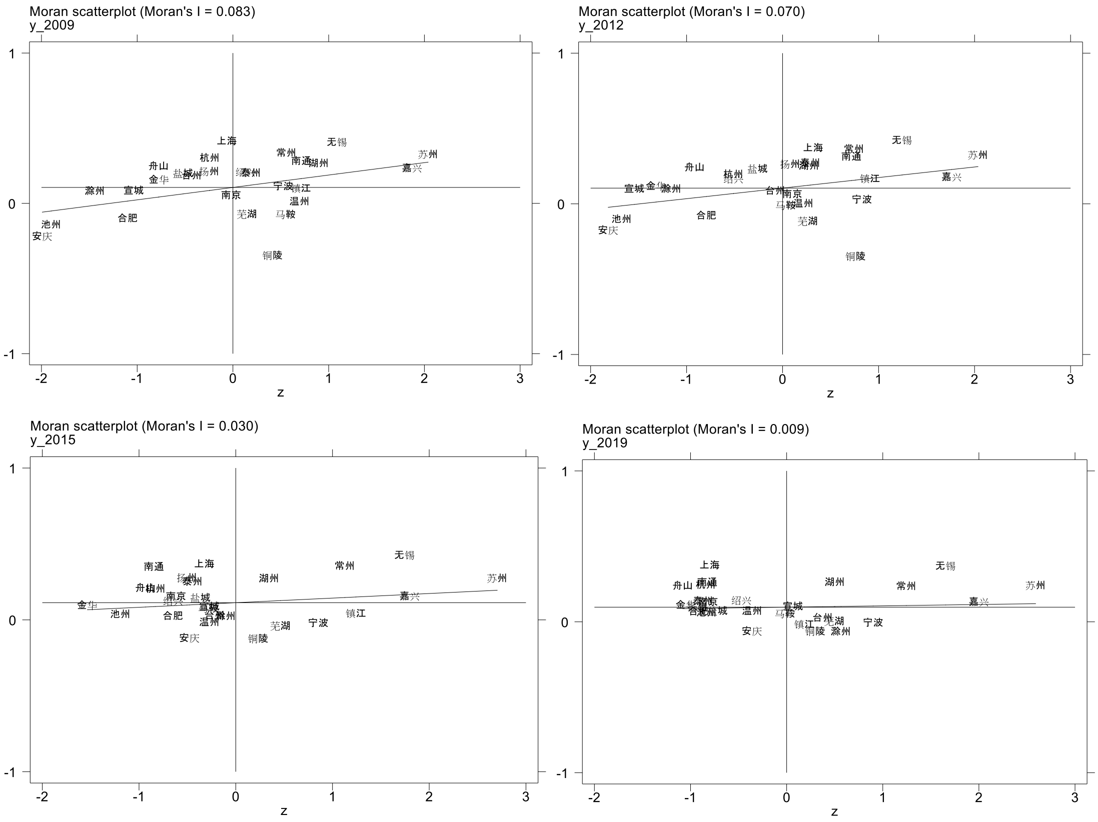

Figure 3. Moran Scatter Diagram of the Collaborative Agglomeration Level of Intercity Manufacturing Industry in the Yangtze River Delta in 2009, 2012, 2015 and 2019.

Figure 3 shows the spatial distribution pattern and dynamic evolution of the collaborative agglomeration level of intercity manufacturing industry in the Yangtze River Delta, further reflecting the spatial correlation and spatial heterogeneity characteristics of the collaborative agglomeration level of manufacturing industry among cities. From the graph, it can be seen that some cities located in the first quadrant, which is known as the "high-high" clustering, are mainly clustered in Jiangsu and Zhejiang provinces, while some cities located in the third quadrant, which is known as the "low-low" clustering, are mainly clustered in Anhui province. This indicates that there is a local high value clustering feature in the collaborative agglomeration level of manufacturing industry in some cities in Jiangsu and Zhejiang,

while some cities in Anhui province have a local low value clustering feature, which is a spatial positive correlation. Overall, the number of cities located in the first and third quadrants continues to decrease, while the number of cities located in the second quadrant, which is the “low-high” value cluster, and the number of cities located in the fourth quadrant, which is the “high-low” value cluster, continue to increase. These cities deviate from the global spatial positive correlation feature and exhibit local negative correlation. As time goes by, the scatter distribution in each quadrant shows a trend of shifting from relative diffusion to relative concentration, indicating that the spatial differences in the collaborative agglomeration level of manufacturing industry between cities are further narrowing, and spatial constraints are gradually being broken.

5. Conclusions and Discussion

The collaborative agglomeration of intercity manufacturing industry is the core content of regional integration. Taking cities in the central area of the Yangtze River Delta as an example, this article collects data on the number of urban unit employees in the manufacturing industry at the end of the period, uses the location entropy index and the modified E-G index to calculate the agglomeration level of manufacturing industry and collaboration level of intercity manufacturing industry in central cities of the Yangtze River Delta. The results show that the manufacturing industry in central cities of the Yangtze River Delta is relatively concentrated. However, the overall agglomeration level is showing a downward trend, with significant intercity differences in the collaborative agglomeration level of manufacturing industry, and a clear trend of transfer of the manufacturing industry to Anhui Province. A spatial weight matrix was constructed based on the mileage of intercity highways. The spatial correlation analysis of the collaborative agglomeration level of intercity manufacturing industry in the Yangtze River Delta was conducted using the Moran’s I and Local Moran’s I indices. It can be seen that the collaborative agglomeration level of intercity manufacturing industry in the Yangtze River Delta has a significant spatial positive correlation, but the overall correlation shows a weakening trend. The spatial differences in the collaborative agglomeration level of manufacturing industry between cities are constantly narrowing.

There is a significant difference in the agglomeration level of manufacturing industry among central cities of the Yangtze River Delta, manifested by the generally high agglomeration level of manufacturing industry in Jiangsu and Zhejiang provinces, while the agglomeration level of manufacturing industry in Anhui province is relatively low. With the transformation and upgrading of the manufacturing industry internally and the external transfer and evacuation, the flow of factors, technology diffusion, and information exchange between cities have influenced the collaborative agglomeration level of manufacturing industry between cities. The spatial differences in the collaborative agglomeration level of manufacturing industry between cities are constantly narrowing, and the trend of cross city and cross province collaborative cooperation in manufacturing industry is becoming increasingly evident. Breaking down the limitations of spatial distance and administrative barriers, promoting the

collaborative agglomeration of intercity manufacturing industry, and accelerating the process of regional integration can be approached from the following three aspects:

Firstly, using urban agglomerations as carriers, strengthen the specialized division of labor and cooperation between cities, and enhance the ability of small and medium-sized cities, especially cities in Anhui Province, to undertake industrial transfer. For cities with high collaborative agglomeration level, it is necessary to accelerate the transformation and upgrading of the manufacturing industry, further strengthen the advantages of manufacturing industry agglomeration, and strengthen and optimize the manufacturing industry. For cities in Anhui Province, it is necessary to selectively undertake the manufacturing industry transferred from Jiangsu and Zhejiang provinces based on their resource endowments and comparative advantages to expand the manufacturing industry, and adhere to the development concept of "center periphery" so as to form different levels of intercity manufacturing division of labor.

Secondly, focus on differentiated development of the manufacturing industry and reduce regional homogeneous competition. Based on the resource endowments and comparative advantages of each city within the urban cluster, it is necessary to clarify their respective manufacturing development priorities combined with the foundation of manufacturing industry development. For cities with good foundation and strong strength, technology intensive industries, especially high-tech manufacturing industry, can be prioritized in the process of manufacturing industry transformation and upgrading to improve the core competitiveness of the manufacturing industry. For cities with poor original infrastructure and the ability to undertake outsourced manufacturing industry, priority can be given to heavy industry in the early stages of undertaking, in order to consolidate the foundation of the manufacturing industry.

Thirdly, break down administrative barriers and spatial boundaries, and strengthen cooperation between governments. In order to achieve regional labor force sharing, technology sharing, and information sharing, it is necessary to strengthen the division of labor and cooperation in the manufacturing industry among cities within the urban agglomeration, improve the integration capacity of industrial resources in the urban agglomeration, as well as promote the free flow of production factors within the urban agglomeration.

no reference? to simple explaining and not covering the results

Author Contributions: Conceptualization, K.Z.; Data curation, W.S.; Formal analysis, W.S.; Funding acquisition, K.Z.; Methodology, K.Z; Visualization, W.S.; Writing—original draft, W.S.; Writing—review and editing, K.Z.. All authors have read and agreed to the published version of the manuscript.

Funding: No funding.

Institutional Review Board Statement: Not applicable.

Informed Consent Statement: Not applicable.

Data Availability Statement: Data used in this study are available upon request.

Conflicts of Interest: The authors declare no conflict of interest.

Reference

1. Wang, X. (2022). High quality development path of Jiangsu's manufacturing industry under the background of Yangtze River Delta integration. Weishi, 432(03), 48-52.
2. Ellison, G. & Glaeser, E. L. (1997). Geographic concentration in U.S. manufacturing industries: A dartboard approach. Political Economy, 105(05), 889-927.
3. Marshall. (1920). Principles of Economics. London: Macmillan and Co., Ltd.
4. Krugman, P. (1991). Increasing returns and economic geography. Political Economy, 3, 483-499.
5. Venables. (1996). Equilibrium location of vertical linked industries. International Economic Review, 2, 341-360.
6. Ellison, G., Glaeser, E. L. & Kerr, W. R. (2010). What causes industry agglomeration? Evidence from co-agglomeration patterns. American Economic Review, 100(03), 1195-1213.
7. Kind, H. J., Knarvik, K. H. M. & Schjelderup, G. (2000). Competing for capital in a “lumpy” world. Publish Economics, 78(3), 253-274.
8. Forslid, R. & Midelfart, K. H. (2005). Internationalisation, industrial policy and cluster. International Economics, 66(01), 197-213.
9. Suedekum, J.(2006). Concentration and specialization trends in Germany since re-unification. Regional Studies, 40(8), 861-873.
10. Kolko, J. & Neumark, D. (2010). Does local business ownership insulate cities from economic shocks? Urban Economics, 67(1), 103-115.
11. Koh, H. J. & Riedel, N. (2014). Assessing the localization pattern of German manufacturing and service industries: A distance-based approach. Regional Studies, 48(5), 823-843
12. Krugman, P. (1992). Geography and Trade. MIT Press.
13. Duranton, G. & Overman, H. G. (2008). Exploring the detailed location patterns of U.K. manufacturing industries using micro-geographic data. Regional Science, 48(01), 213-243.
14. Lu, J. (2014). Research on the collaborative effect of productive services based on manufacturing agglomeration. Journal of Management, 11(03), 396-401.
15. Sheng, F. (2014). Agglomeration of productive services and upgrading of manufacturing industry: mechanism and experience: spatial econometric analysis from 230 City Data. Industrial Economy Research, 69 (02), 32-39+110.
16. Wang, Y., Sun, C. (2019). The impact of industrial collaborative agglomeration on industrial structure optimization: an empirical analysis based on high tech industries and productive service industries. Exploration of Economic Issues, 447(10), 146-154.
17. Tang, C., Qiu, J., Zhang, L., et al. (2021). Spatial econometric analysis of the impact of factor flow and industrial collaborative agglomeration on regional economic growth: A case study of manufacturing and productive service industries. Economic Geography, 41(07), 146-154.
18. Liu, Y., Liu, B. (2016). The impact of collaborative agglomeration of producer services and manufacturing on manufactur-ing efficiency: an empirical study based on panel data of China's urban agglomeration. Economic Management, 38(06), 16-28.
19. Ryan, M. (2013). Gallagher shipping costs, information costs, and the sources of industrial co-agglomeration. Regional Science, 53(02), 304-331.
20. Gabe Todd, M. & Abel Jaison, R. (2013). Shared knowledge and the co-agglomeration of occupations, staff report. Federal Reserve Bank of New York, Working article No.612.
21. Yang, R. (2015). Can industrial agglomeration improve environmental pollution in China. China Population, Resources and Environment, 25(02), 23-29.
22. Zhang, Z., Qin, S. (2018). The impact of industrial agglomeration on urban green efficiency: a case study of 108 cities in the Yangtze River Economic Belt. Urban Issues, 276(07), 48-54.
23. Chen, X., Zhu, J. & Li, G. (2018). Regional differences and influencing factors of collaborative agglomeration among manufacturing industries in China. Economic Geography, 38(12), 104-110.
24. Ciccone, A. & Hall, R. E. (1996). Productivity and the density of economic activity. The American Economic Review, 86(1), 54-70.

25. Gaulier, G., Lemoine, F., etc. (2007). China’s integration in east Asia: Production sharing, FDI and high-tech trade. Economic Change and Restructuring, 40(12), 27-63.
26. Ke, S. (2010). Agglomeration, productivity, and spatial spillovers across Chinese cities. The Annals of Regional Science, 45(1), 157-179.
27. Keeble, D., Wilkinson, F., etc. High-technology clusters, networking and collective learning in Europe. Aldershot: Ashgate, 2000.
28. Holmes, T. J. (2010). Structural， experimentalist， and descriptive approaches to empirical work in regional economics. Regional Science, 50(01), 5-22.
29. Combes, P. P. (2000). Economic structure and local growth: France, 1984-1993. Urban Economics, 47(03), 329-355.
30. Park, S.H. (1994). Intersectoral relationships between manufacturing and service: New evidence from selected pacific basin countries. ASEAN Economic Bulletin, 14(03), 245-263.
31. Andersson, M. (2004). Co-location of manufacturing & producer services——A simultaneous equation approach. CESIS Electronic Working Paper Series Paper.
32. Chen, J., Liu, Y. & Zou, M. (2016). Improving urban production efficiency under industrial collaborative agglomeration: based on the background of integration innovation and development power transformation. Journal of Zhejiang University (Humanities and Social Sciences Edition), 46(03), 150-163.
33. Zhang, H., Han, A. & Yang, Q. (2017). Spatial effect analysis of collaborative agglomeration between manufacturing and productive services in China. Quantitative Economic and Technological Economic Research, 34(02), 3-20.
34. Anselin L. (1998). A test for spatial auto-correlation in seemingly unrelated regressions. Economics Letters, 28(04), 335-341.
35. Elhorst, J. P. & Freret, S. (2009). Yardstick competition among local governments: French evidence using a two-regimes spatial panel data model. Regional Science, 49(02), 931-951.
36. Fuscom, E. & Vidoli, F. (2013). Spatial stochastic frontier models: Controlling spatial global and local heterogeneity. International Review of Applied Economic, 27(05), 679-694.
