# Supplementary material for: Collaborative agglomeration level and spatial correlation of intercity manufacturing industry: An empirical study based on the cities of the Yangtze River Delta
Source: PLoS One. 2023 Nov 10;18(11):e0293645. doi: 10.1371/journal.pone.0293645 (PMC10637707; doi:10.1371/journal.pone.0293645)
Supplement: Attachment — Submitted filename: Response to Reviewers.docx [file pone.0293645.s003.docx]

Response to Reviewers

Dear editorial department and reviewers, thank you very much for your review and valuable comments. We have revised the article according to your comments. The relevant modifications are briefly described as follows:

**Point 1:** A figure of the research objects should be provided in section 2.1.1 so that people could have a sense of where the Yangtze River Delta is.

**Response 1:** Due to the fact that the location map of the Yangtze River Delta may involve maps/satellite images which may be copyrighted, we have provided a textual description of the approximate location of the Yangtze River Delta as an alternative in the text section. Details are shown in the figure below:

**Point 2:** The data source needs to be explicitly described.

**Response 2:** In section 2.2.1, we provided a detailed description of the data sources. Details are shown in the figure below:

**Point 3:** Texts in Figure 3 should be changed to English.

**Response 3:** We have made modifications to Figure 3 by replacing Chinese with English. Details are shown in the figure below:

**Point 4:** Policy implications from empirical findings should be discussed at the end.

**Response 4:** In the conclusion and discussion section, we supplemented the policy implications from empirical findings. Details are shown in the figure below:

Dear editorial department, we have made modifications to the points that were requested in your last email. However, due to the 'An unmarked version of revised paper without tracked changes' upload error when we submitted the revised version of the manuscript in the system, we would like to provide the following explanations for the five issues you raised in this email:

**Issue 1:** We note that Figure 2 in your submission contain map/satellite images which may be copyrighted. All PLOS content is published under the Creative Commons Attribution License (CC BY 4.0), which means that the manuscript, images, and Supporting Information files will be freely available online, and any third party is permitted to access, download, copy, distribute, and use these materials in any way, even commercially, with proper attribution. For these reasons, we cannot publish previously copyrighted maps or satellite images created using proprietary data, such as Google software (Google Maps, Street View, and Earth).

**Response 1:** To avoid copyright issues, we have removed Figure 2 and replaced the illustrations with textual descriptions in the corresponding parts of the main text. Details are shown in the figure below:

**Issue 2:** We note that the grant information you provided in the ‘Funding Information’ and ‘Financial Disclosure’ sections do not match.

When you resubmit, please ensure that you provide the updated ‘Financial Disclosure; we will change the online submission form on your behalf.

This information should be included in your cover letter; we will change the online submission form on your behalf.

**Response 2:** We have added “Funding Information” to the revised cover letter, and we will provide the updated "financial disclosure" when resubmitting the manuscript in the system. Details are shown in the figure below:

**Issue 3:** Please amend the title either on the online submission form or in your manuscript so that they are identical.

**Response 3:** We have amend the title on the online submission form. However, due to the 'An unmarked version of revised paper without tracked changes' upload error when we submitted the revised version of the manuscript in the system, the title is inconsistent. We confirm that the title either on the online submission form or in our manuscript this time are identical.

**Issue 4:** Your abstract cannot contain citations. Please remove any existing citations from the abstract section. You may only include citations in the body text of the manuscript, and please ensure that they remain in ascending numerical order on first mention.

**Response 4:** Due to the use of numerical symbols in the abstract to illustrate the research conclusions, there is a divergence in whether it is a citation annotation. To avoid disagreements, we have removed numerical symbols from the abstract and expressed them in English. Details are shown in the figure below:

**Issue 5:** Please include a copy of Table 2 which you refer to in your text.

**Response 5:** Due to naming errors, we named Table 2 as Table 3. We have made revisions in the manuscript. Details are shown in the figure below:
